# Leveraging Technology for the Wellbeing of Individuals With Autism Spectrum Disorder and Their Families During Covid-19

**DOI:** 10.3389/fpsyt.2021.566809

**Published:** 2021-06-28

**Authors:** Ceymi Doenyas, Samaa M. Shohieb

**Affiliations:** ^1^Research Center for Translational Medicine, Koç University, Istanbul, Turkey; ^2^Faculty of Computers and Information, Mansoura University, Mansoura, Egypt

**Keywords:** applications (“Apps”), autism spectrum disorder, COVID-19, education technologies, technology

## Introduction

Recently, the need for a mental health technology revolution during COVID-19 was noted (1). These authors suggested that interventions should be targeted toward vulnerable groups and adapted to their individual needs (1), and we have expanded this concept here. One vulnerable group that will benefit from such a technology revolution and targeted interventions comprises individuals with Autism Spectrum Disorder (ASD). We discuss how technology can be leveraged to address the specific challenges experienced by these individuals and their families during the pandemic.

## Using Technology for Families With ASD During the Pandemic

One way technology can be used during the pandemic for families with ASD is through telehealth, which refers to providing various remote services electronically, such as patient care, education, and monitoring (2). Relatedly, telemental health is the utilization of information and communication technologies to remotely provide mental health care, evaluations, and therapy (3). Telemental health is viewed as a valuable tool during COVID-19 as it can effectively respond to the mental health needs of individuals in isolation or with restricted mobility while minimizing infection risk and, therefore, can be an option to provide care without interruptions and with adherence to social distancing (3).

### Remote Intervention Administration to Parents and Children

Attempts at using technology for remote service delivery to families with ASD predate the pandemic. One study focusing on parent coaching instead of a direct intervention with children with ASD showed that parents, service providers, and ASD specialists perceived remote technologies to be helpful by improving the skills of the parents; reducing cost, time, and travel; providing flexible, ongoing, and regular support; and allowing families to access support from their home. At the same time, this remote coaching often resulted in frustration due to technical difficulties, and it was agreed that remote technology should not replace face-to-face contact but only augment it (4). Therefore, overcoming such frustrations to harness the provided advantages will be important in refining remote parent coaching for families with ASD.

Another study exploring this possibility before the pandemic focused on web-based training and telemedicine to train parents to implement the Applied Behavior Analysis (ABA) strategies with their children. This program was beneficial in increasing parents' knowledge of the ABA strategies and their implementation (5). These findings are corroborated by a systematic review of a remote parent-mediated intervention training yielding a preliminary evidence of improved parental knowledge and social behavior and communication skills of children with ASD (6). In terms of the remote interventions for children with ASD, one hopeful finding shows that children with ASD who received remote support and those who received face-to-face support did not significantly differ in terms of the gains they made in terms of social communication as measured by initiations of joint attention and requesting (7). Such protocols can be exploited during COVID-19 to remotely provide interventions to children with ASD and to provide training to their parents to implement validated intervention strategies at home.

A web-based parent training tutorial to enhance interactions was found by parents to be user-friendly and easy to understand. They reported that the tutorial increased their knowledge about how to communicate with their child and that they felt comfortable to apply these techniques to their communication with their child (8). Another program for parents included live distance coaching sessions, in addition to online activities and interactive tutorials, on how to use the ABA procedures to teach new skills, generalize them to other settings, and reduce challenging behavior (5). This program resulted in gains in the parents' knowledge and ABA implementation skills that were independent of their educational background.

One instance of telemental health administration for families of individuals with ASD was recently started in Italy by a group of professionals *via* online observations and discussions with families they had been consulting with since before the lockdown (9). They coached parents on structuring the entire day for their children with ASD and their siblings, selecting contextually appropriate activities, and setting up a positive reinforcement system at home. The researchers observed that many parents were able to implement these effectively, bring order to their homes, and help their children be happy, calm, productive, and engaged (10).

Using remote intervention techniques for families with ASD to supplement face-to-face interventions would be ideal, as would using both online tutorials and live distance coaching sessions. Yet, when the available resources do not allow for these ideal conditions, using web-based tutorials to disseminate knowledge to parents of children with ASD can provide them with resources to more successfully handle the difficulties of pandemic conditions for their families.

### Remote Psychological Counseling

In addition to providing interventions and parent training remotely, technology may enable remote psychological counseling for families with ASD. Because of various limitations that prevent these families from receiving full-time psychological services, it has been suggested that remote counseling may emerge as the only available alternative support for some families of children with ASD (9). For a successful online counseling for these families, it has been recommended that the family environment of the child should be taken into account, the consultation provider should be a professional, interventions should have measurable characteristics, and face-to-face interactions with the child with ASD should be possible (9). Though these recommendations may better apply to non-pandemic conditions, they can nonetheless be established during COVID-19 as well by using videoconferencing for the face-to-face interactions and ensuring the other conditions for the counseling process. It has been noted that, although, ideally, teletherapy should not replace in-person services, it does become necessary when no other comparable service option is available (11), which may be the case during the pandemic for many families with ASD.

### Social Connectedness

Another way technology can be used during the lockdown is for social connectedness. There is evidence from a sample of adults with ASD that the majority of that sample of 108 adults used social networking sites, and the most commonly given reason for this was for social connection. However, decreased loneliness was not specifically associated with social media use but was associated with offline friendship's quality and quantity (12). In line with this, during the pandemic conditions, the ability and prevalence of social media utilization by individuals with ASD should not be assumed to be enough to alleviate their feelings of loneliness. Therefore, other programs geared toward fostering virtual interactions could be implemented by autism communities and foundations to assist with forming such connections.

### Mobile Applications

Embracing the suggestion that digital mental health tools should be affordable, accessible, and appropriate for all individuals (1), we discuss how technological applications can be used to address two fundamental adversities faced by individuals with ASD during COVID-19: understanding and following the measures necessitated by COVID-19 and continuing special education during the implemented lockdown and social distancing measures.

Firstly, using technology to help individuals with ASD comprehend and follow the COVID-19-related measures is important, as our study showed that 75% of the parents of children with ASD reported that their children did not understand properly, or understood only to a medium degree, the COVID-19-related measures and necessities, such as staying home or social distancing (13). When asked if they were using a resource with their children that explained what COVID-19 is and what needs to be done, 80% said no, and 85% said that they would want such a resource for children with ASD and if it existed, they would use it (13). For this purpose, our Crises and Disasters Management Game (CDMG) (14) can be adapted following the guidelines for developing computer games for children with ASD (15). CDMG is a simulation game for man-made crises (e.g., fires) and natural disasters (e.g., earthquakes). In this game, the players lose points for engaging in behaviors that can cause or exacerbate crises. By experiencing different scenarios, such as that of an indoor fire, players learn how to safely navigate through crises and disasters. Being a simulation game, CDMG is a safe and cost-effective choice that presents no danger or risk to players. Given these properties, CDMG can be adapted to help children with ASD keep safe during the COVID-19 pandemic. For instance, the new addition of a COVID-19 safety precautions scenario can teach children with ASD how to take personal protection precautions such as staying home and washing their hands. Children with ASD may, in turn, gain points and receive reinforcers as they do so, which are the commonly used behavior modification methods in special education.

**Figure 1:**
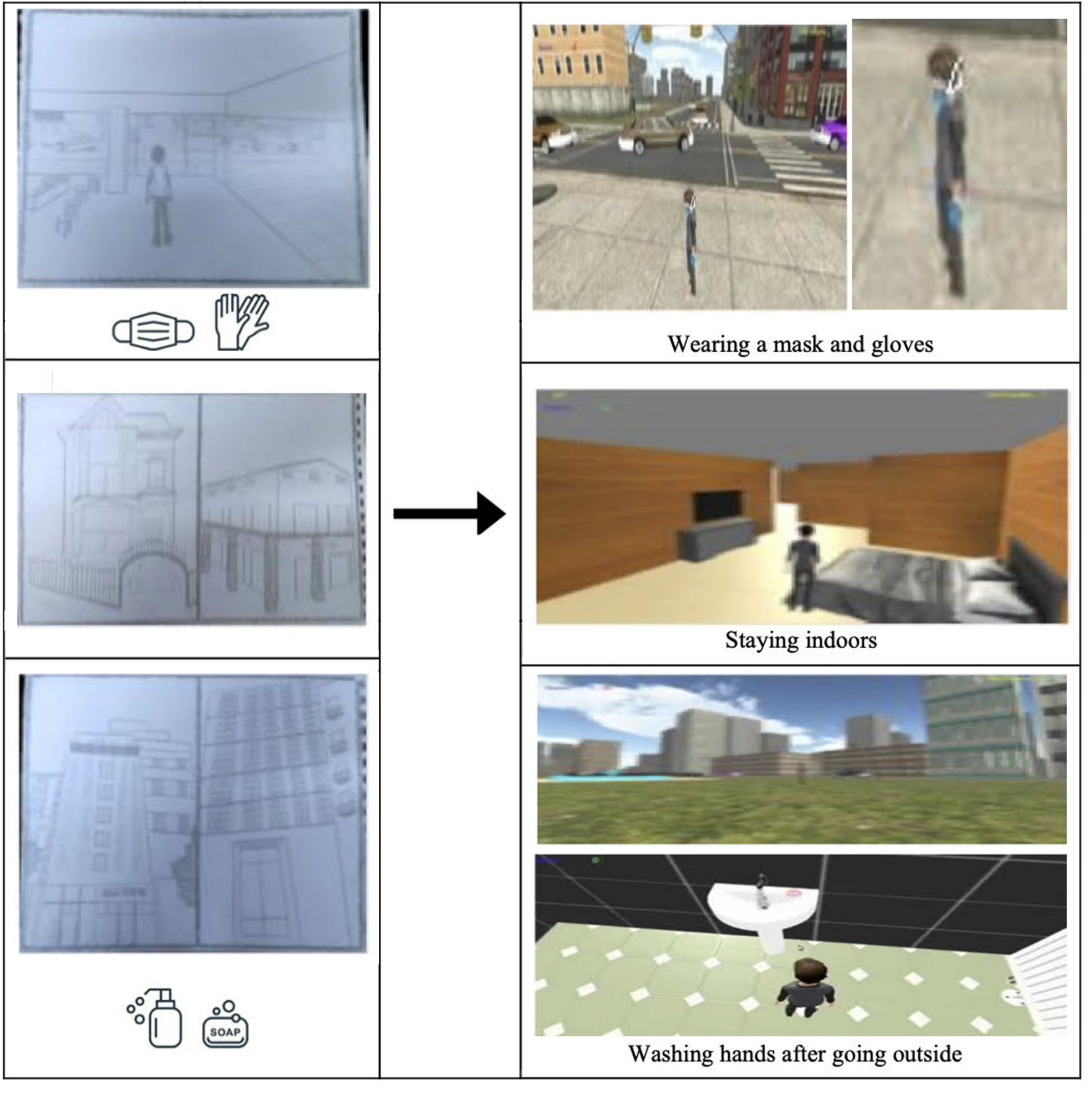
From hand modelling to graphical user interface (GUI) design of a potential application or module for teaching Covid-19 measures to children with autism, adapted from (3).

Another scenario can simulate safety measures outside, where children can select to use masks, gloves, and other hygienic products, such as alcohol-based disinfectants, and gain points as they abide by these measures (see Figure 1). The rules for designing softwares for children with ASD (15) can easily be implemented in the CDMG game. For instance, since children with ASD prefer reliable routines and predictable environments, the game interface can be kept as simple as possible with a low differentiation between global and local cohesion. Such accommodations can make the new scenarios added to this game to be ASD-friendly and present a safe and fun environment for them to practice the COVID-19-related measures, which will reinforce these behaviors with rewards and points and increase their likelihood of being applied in real life.

Secondly, special education for individuals with ASD is halted due to the lockdown and social distancing measures necessitated by the pandemic. Although mainstream education can continue *via* distance education for typically developing individuals, this is not the case for the one-on-one, special education for individuals with ASD. Our findings revealed that 92% of the children with ASD are not continuing their special education during COVID-19 (13). Therefore, in addition to the telemental health opportunities it offers, technology enables individuals with ASD to continue their special education from home with applications created specifically for them using the ABA principles that are used in their special education centers.

In 2014, we designed an application that teaches children with ASD a skill that is part of the special education curriculum *via* tablets (16). The ABA-based hinting and scoring system we created was used as the basis for the autism education application Otsimo, which was selected as the best autism education application of 2019 by the University of Edinburgh. Otsimo now has a total of 339,989 users from 180 countries; of which, 208,176 use it for special education and the rest for speech therapy. Though this application initially targeted the ASD population, it is now being used by other developmental disability groups as well, serving as a technological platform to remotely address the educational and speech therapy needs of those with speech delay, developmental delay, ADHD, apraxia, premature birth, cerebral palsy, and Down syndrome. More recently, we created an ABA-based language teaching application for individuals with ASD using dynamic difficulty adjustment principles, which increased the engagement of children by adapting the content to their skill levels and the time spent with educational materials at home for these families^1^. Therefore, technology can also be used to bring special education to the homes of families with ASD (17).

## Conclusion

We believe that the recently recommended mental health technology revolution (1) should include components specifically designed for individuals with ASD who experience behavioral and social challenges even without the strains of the COVID-19 period. Therefore, we recommend that the authors' suggestion of promptly investing in high-quality and accessible online and mobile mental health technologies during this pandemic (1) should include applications for disaster protocol training and distance education specifically geared toward individuals with ASD to help make this taxing period more manageable for the affected individuals and their families. Additionally, previous evidence and reports about using technology for remote parent training, for interventions for children with ASD, and for counseling for families with individuals with ASD can guide authorities to adapt such services to the pandemic conditions. We suggest that this could be done by formulating effective telemental health options that overcome the challenges reported for these remote endeavors in the past, and take into account the difficulties disclosed by the parents of children with ASD and sources for which they communicate a need in order to better cope with the changes associated with lockdowns and other measures necessitated by the pandemic.

## Author Contributions

CD conceived and designed the manuscript and completed the revisions. CD and SS wrote the manuscript together. SS created the figure. All authors read and approved the submitted version.

## Conflict of Interest

The authors declare that the research was conducted in the absence of any commercial or financial relationships that could be construed as a potential conflict of interest.

## Footnotes

^1^Shohieb S, Doenyas C, Elhady AM. Dynamic difficulty adjustment technique-based mobile vocabulary learning game for children with autism spectrum disorder. (Under review).
